# Corrigendum: Novel variation at chr11p13 associated with cystic fibrosis lung disease severity

**DOI:** 10.1038/hgv.2017.16

**Published:** 2017-05-25

**Authors:** Hong Dang, Paul J Gallins, Rhonda G Pace, Xue-liang Guo, Jaclyn R Stonebraker, Harriet Corvol, Garry R Cutting, Mitchell L Drumm, Lisa J Strug, Michael R Knowles, Wanda K O’Neal

**Correction to:** *Human Genome Variation* (2016), **3**, doi: 10.1038/hgv.2016.20; advance online publication 7 July 2016

After the online publication of this article, the authors noticed an error in the figure and legend of Figure 2.

The correct figure should show that leftmost SNP is rs7939918.

The correct Figure 2 legend statement of this article should have read in next page.

The authors apologize for any inconvenience caused.

## Figures and Tables

**Figure 2:**
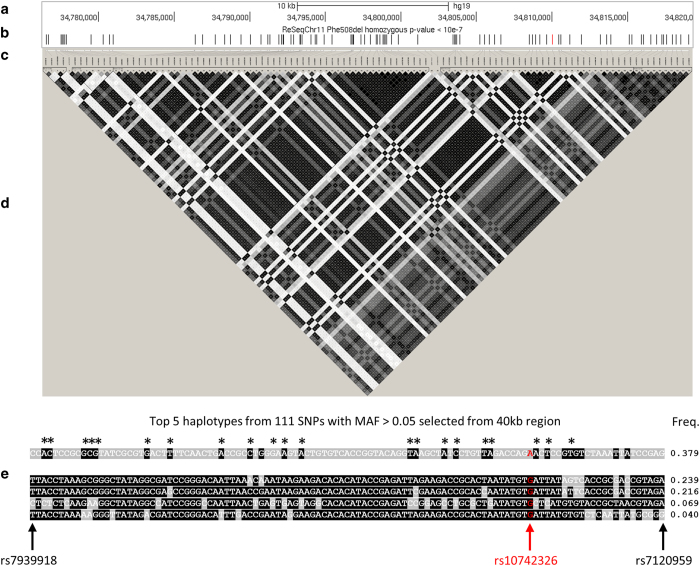
Linkage disequilibrium (LD) and haplotype structure around top cystic fibrosis (CF) lung disease-associated region. The upper panels **a–d** show the entire CF lung disease severity association region, whereas the lower panel **e** indicates the LD structure and the top 5 haplotypes of single-nucleotide polymorphisms (SNPs) with the most significant association *P* value (<10^−7^). The sections are: (**a**) scale bar and genome coordinates on chr11 of University of California Santa Cruz (UCSC) hg19 reference genome; (**b**) CF lung disease severity association *P* values; (**c**) LD plot SNP locations with respect to the genome coordinates in **a** (upward tick marks) that are then mapped to the LD plot in **d** (slanted lines); (**d**) LD plot generated by Haploview; (**e**) haplotype structure with allele genotypes and frequencies; the first SNP (rs7939918) and last SNP (rs7120959) are labeled in black font with black arrows at the bottom; the SNP of highest significance (rs10742326) is labeled in red font with a red arrow. Asterisks (*) indicate common alleles observed in the top five haplotypes.

